# Evaluation of cadmium and arsenic effects on wild and cultivated cardoon genotypes selected for metal phytoremediation and bioenergy purposes

**DOI:** 10.1007/s11356-021-14705-9

**Published:** 2021-06-15

**Authors:** Chiara Leonardi, Valeria Toscano, Claudia Genovese, Julian Frederick Willem Mosselmans, Bryne Tendelo Ngwenya, Salvatore Antonino Raccuia

**Affiliations:** 1 Department of Biological, Geological and Environmental Sciences, University of Catania, via Androne, 81, 95124 Catania, Italy; 2 National Research Council, Institute for Agricultural and Forest Systems in the Mediterranean, Via Empedocle, 58, 95128 Catania, Italy; 3 Diamond Light Source Ltd, Diamond House, Harwell Science and Innovation Campus, Fermi Ave, Didcot, OX11 0DE UK; 4 School of GeoSciences, University of Edinburgh, Drummond St, Edinburgh, EH8 9XP UK

**Keywords:** Heavy metal(loid)s, *C. cardunculus* var. *altilis*, *C. cardunculus* var. *sylvestris*, Detoxification mechanisms, Speciation distribution

## Abstract

*Cynara cardunculus* L. is a multipurpose crop, characterized by high production of biomass suitable for energy purposes and green chemistry. Taking advantage of its already demonstrated ability to grow in polluted environments that characterize many world marginal lands, the aim of this work was to investigate the response of different cardoon genotypes to exposure to cadmium (Cd) and arsenic (As) pollution, in order to use this crop for rehabilitation of contaminated sites and its biomass for energy production. In this study, seeds of two wild cardoon accessions harvested in rural and industrial Sicilian areas and of a selected line of domestic cardoon were used, and the grown plants were spiked with As and Cd, alone or in combination, at two different concentrations (500 and 2000 μM) and monitored for 45 days. The growth parameters showed that all the plants survived until the end of experiment, with growth stimulation in the presence of low concentrations of As and Cd, relative to metal-free controls. Biomass production was mostly allocated in the roots in As treatment and in the shoots in Cd treatment. Cd EXAFS analysis showed that tolerance to high concentrations of both metals was likely linked to complexation of Cd with oxygen-containing ligands, possibly organic acids, in both root and leaf biomass with differences in behaviour among genotypes. Under As+Cd contamination, the ability of the plants to translocate As to aboveground system increased also showing that, for both metal(loid)s, there were significant differences between genotypes studied. Moreover, the results showed that *Cynara cardunculus* var. *sylvestris* collected in an industrial area is the genotype that, among those studied, had the best phytoextraction capability for each metal(loid).

## Introduction

Heavy metals and metalloids pollution is a major environmental and human health problem in all industrialized countries resulting from industrial activities, modern agricultural practices and mining (Adriano 2001; Miguel and Marum 2011; Pérez-Sirvent et al. 2012; Fernández et al. 2013; Guarino et al. 2018; Sahito et al. 2021). Among metals and metalloids, of most concern are As and Cd; both are highly toxic and have no known physiological benefit. As toxicity is implicated as a probable cause of bladder, lung, skin and prostate cancer in humans, among others (Peralta-Videa et al. 2009). Meanwhile, Cd can be absorbed via the alimentary tract, penetrates through placenta during pregnancy, and damages membranes and DNA (Kabata-Pendias 2004). Moreover, Cd may cause kidney and bone damage, affects the female reproduction system, which implies a serious threat for mammals and humans (Peralta-Videa et al. 2009), and is the only metal that might create human or animal health risks at plant tissue concentrations that are not generally phytotoxic (Peijnenburg et al. 2000).

Although high concentrations of trace elements in agro-ecosystems influence the growth and development of the plants through negative impacts on processes such as respiration, photosynthesis, electron transport and cell division (Wójcik et al. 2009; Pourrut et al. 2011; Muszyńska and Hanus-Fajerska 2015), different plant species are able to tolerate them, survive, grow, and reproduce on soils contaminated with heavy metals and metalloids (Muszyńska and Hanus-Fajerska 2015). This is thought to occur through a variety of mechanisms, including storage and detoxification/sequestration of heavy metals and metalloids (Tran and Popova 2013) in the shoot, mainly based on chelation and subcellular compartmentalization (Yadav 2010; Tran and Popova 2013) or maintaining shoot concentrations at low level up to a critical soil value above which relatively unrestricted root-to-shoot transport result (Violante et al. 2010). Phytoremediation is a biological technique that uses such plants to remediate soils contaminated with trace metals; the choice of plant depends on a variety of factors, including high biomass production and high metal tolerance.

Among the species proposed to remediate the soils from metal(loid)s, there is a growing interest on *Cynara cardunculus* L. (cardoon), a perennial species from Asteraceae family, native to Mediterranean countries. It comprises the subspecies, *C. cardunculus* L. subsp. *scolymus* (L.) Hegi = *C. cardunculus* L. subsp. *scolymus* (L.) Hayek (globe artichoke) and two botanical varieties *C. cardunculus* L. var. *altilis* DC. (domestic cardoon) and *C. cardunculus* L. var. *sylvestris* Lam. (wild cardoon) that is considered to be the wild ancestor of globe artichoke (Rottenberg and Zohary 1996; Raccuia et al. 2004a). The domestic cardoon has been cultivated for many years as a traditional food source in some parts of southern Europe, particularly in Italy, France and Spain. In addition, its high production of biomass and grain (Raccuia and Melilli 2007; Angelini et al. 2009; Raccuia et al. 2012) can be used for different purposes, including feed, bioenergy, green chemistry, pharmaceutical, nutraceutical and phytoremediation of heavy metals (Raccuia and Melilli 2004; Genovese et al. 2016a, 2016b; Leonardi et al. 2016a, 2016b; Raccuia et al. 2016; Toscano et al. 2016; Gominho et al. 2018). The wild cardoon is a robust thistle with a characteristic rosette of large spiny leaves and branched flowering stems that accumulate biomass mainly in roots (Raccuia and Melilli 2004).

All these characteristics, its good adaptability to the Mediterranean climate, to stressful environmental conditions (salt, heat and drought stress) (Mauromicale and Licandro 2002; Raccuia et al. 2004b; Benlloch-González et al. 2005; Argento et al. 2016; Docimo et al. 2020; Pappalardo et al. 2020), its good tolerance to stress induced by contaminants both during germination and growth phases (Llugany et al. 2012; Sánchez-Pardo et al. 2015; Leonardi et al. 2016a, 2016b; Pappalardo et al. 2016, 2020; Arena et al. 2017; Capozzi et al. 2020) and low input management, suggested its potential use for phytoremediation. From these studies, the potential of cardoon to accumulate heavy metals and metalloids from polluted soils is very clear. However, to date there are no specific studies regarding the resistance mechanisms to pollutants, of different varieties of cardoon, and whether there is influence of the genotype. These studies could be useful to understand at the same time the interesting dual use of this crop not only to remediate contaminated soils from toxic elements but also for biomass production for bioenergy (Mehmood et al. 2017). The energy potential of cardoon is attributable not only to the characteristics listed above but also to low moisture content of the biomass at harvest; a biomass composition mainly of a lignocellulosic-type and a high heating value (Fernández et al. 2006). Toscano et al. (2016) carried out two different pilot systems for biodiesel and pellet productions using cardoon biomass and grain: the results showed that cardoon plants may be used for different energetic purposes, making cardoon a very competitive and sustainable energy crop in Mediterranean environment and an economic alternative for farmers. In fact, from the perspective of the circular economy, the Asteraceae family and some members of the families Brassicaceae, Poaceae, Fabaceae and Malvaceae are fast-growing economic crops, and their biomass production during phytoremediation activities will make an increasing contribution to meet sustainable future energy demands (Ingrao et al. 2016; Witters et al. 2012; Sahito et al. 2021; Zehra et al. 2020a; Zehra et al. 2020b).

In this work, the growth under As and Cd stress conditions of two varieties of wild cardoon and one domestic cardoon were compared with the aim to (i) evaluate the variability in response of different varieties and genotypes of cardoon plants; (ii) assess the concentration, bioaccumulation and translocation ability of As and Cd in different parts of the plant; and (iii) understand the tolerance of these plants to heavy metal(loid)s.

## Materials and methods

### Plant material

For this research, we used different cardoon genotypes, belonging to the genetic bank of the National Research Council-Institute for Agricultural and Forest System in the Mediterranean city of Catania, Sicily-IT (CNR-ISAFOM): one *C. cardunculus* L. var. *altilis* (Gen.1) and two genotypes of *C. cardunculus* L. var. *sylvestris* Lam. (Gen.2 and Gen.3). The domestic cardoon (*C. cardunculus* var. *altilis* DC.) is a selected line by CNR-ISAFOM to produce biomass for green chemistry. The wild cardoon (*C. cardunculus* var. *sylvestris* Lam.) populations were collected in two different sites in Eastern Sicily (Raccuia et al. 2004a). The first (R14CT—Gen.2) was collected at 820 m above sea level in Randazzo (CT-Sicily-IT) (37° 53′10.9″ N 14° 57′13.2″ E), within Nebrodi Regional Park. The second (A14SR—Gen.3) was collected in the territory of Augusta (SR-Sicily-IT) (37° 14′13″ N 15° 11′05″ E) at 2 m above sea level. The two wild genotypes were chosen precisely because they came from two very different sites: the R14CT genotype was selected in a high mountain area characterized by an uncontaminated environment, temperatures ranging from a minimum of 3 to a maximum of 28 °C and an average annual rainfall of 830.5 mm; the A14SR genotype, on the other hand, was selected from a coastal and industrial area near the port city of Augusta (SR). This site is characterized by temperatures ranging from 7 to 30 °C and an average annual rainfall of 714.1 mm (SIAS - Sicilian Agrometeorological Information Service - SIAS – Sicily n.d.). All the cardoon populations were harvested during the summer of 2014, and both the wild genotypes used for our trials were collected in uncultivated lands within patches of cardoon spontaneous vegetation.

### Field experimental design

To simulate field conditions, the trial was conducted outdoors in controlled environmental conditions in CNR-ISAFOM experimental field located at Cassibile, Syracuse (Sicily) (36° 58′33″ N 15° 12′17″ E) at 50 m above sea level from December 2014 (sowing) to July 2015 (last harvested). The monthly minimum temperatures ranged from 4 in February to 20.5 °C in July and the maximum ones from 16.6 in December to 34.3 °C in July; the average annual rainfall was 555.1 mm (SIAS - Sicilian Agrometeorological Information Service - SIAS – Sicily n.d.). For the sowing experiment, 396 seeds of Gen.1, Gen.2 and Gen.3 were placed in plastic pots (diam. 3 cm) using a clear cover to retain moisture until seedlings appeared, to allow selecting healthy seedlings.

In January 2015, 4-week-old wild and domestic cardoon plants with three or four leaves were transplanted into plastic pots (diam. 45 cm) filled with 13.0 kg of commercial potting soil (1 plant per pot, 3 independent biological replicates, 189 plants in total). After 2 weeks of planting, a N-P-K fertilization (20:10:10) was added to the soil with a ring application at the rate of 50 g per pot, and it was repeated each 30 days at the same rate until the end of the experiment. Five months after sowing, 500 mL of aqueous solution of As, Cd or As+Cd, each at two different concentrations 500 and 2000 μM, were added to each pot and compared with a control. The experimental design sought to vary the concentrations to reflect high metal stress conditions but also low stress conditions where growth might be stimulated through hormesis. The actual concentrations were chosen based on preliminary trials (Leonardi et al. 2016a, 2016b; Pappalardo et al. 2016), which indicated differences in plant response over these concentrations and by comparison with values used in the literature (Sun et al. 2008; Papazoglou 2011; Llugany et al. 2012). The arsenic solutions, created from sodium dibasic arsenate heptahydrate (Na_2_HAsO_4_ 7H_2_O), and those of cadmium, made from cadmium nitrate tetrahydrate (Cd (NO_3_)_2_ 4H_2_O), were named as As0 (control), As500 and As2000, and Cd0 (control), Cd500 and Cd2000, respectively, with the numbers representing concentration in micromolar. Finally, the mixtures of both metals were prepared by concentrated solutions of As and Cd and named as As0+Cd0 (control), As500+Cd500 and As2000+Cd2000.

The pots within each treatment were arranged adopting a randomized experimental design. Crop water requirements were satisfied by a drip irrigation system with a flow control valve which made it possible to reduce percolation losses. During the growth cycle, plant growth parameters (height, number of leaves) and visual systems such as the presence of yellow and dried leaves were recorded, and each individual plant was observed, in order to detect visible toxicity symptoms.

### Sampling and chemical analysis

Cardoon plants (3 plants per treatment) were harvested at 3 different time points during growth, every 15 days until 45 days after the artificial contamination of the soil, from June to July 2015. After the harvest, plants were gently removed from the pots, and the fresh weights of the individual plant per genotype and treatment were subsequently determined. Shoots and roots were further separated, and after removing the soil particles from roots, they were washed with tap water, then with distilled water and finally with 0.01 M HCl for approximately 5 s in order to remove external metals from the root surface (Gardea-Torresdey et al. 2004). Root length (cm) and dry biomass of roots and shoots were determined. Finally, the samples were dried for 72 h in a temperature-controlled oven at 70 °C. Soil samples were collected from each pot, air-dried at room temperature and ground to pass a 2.0-mm mesh.

For chemical analysis, samples of roots and shoots were cut with stainless steel scissors and ground in an agate pestle and mortar with liquid nitrogen to obtain homogeneous samples. The powdered dry plant samples were digested in a closed-vessel microwave digestion system (MARSXPRESS by CEM Corporation, NC, USA) equipped with sensors for temperature and pressure (175 °C, 1600 W). Triplicate 0.5 g samples with 1 mL of Yttrium internal standard (1 mg L^−1^) were put inside the microwave vessels and digested in a mixture of 8 mL of 65% HNO_3_ and 2 mL of 30% H_2_O_2_. After digestion, the solution was quantitatively transferred into pre-cleaned 50-mL volumetric flasks and diluted to the mark with deionized water. The samples were stored at 4 °C for subsequent analysis. The concentration of As and Cd in soil samples was determined by triplicate digestion of 0.5 g soil sample in a high pressure microwave system (175 °C, 1600 W) with a mixture of 3 mL 65% HNO_3_ and 9 mL 37% HCl (USEPA 3051A-USEPA 1998). After digestion, the solution was quantitatively transferred into pre-cleaned 50-mL volumetric flasks and diluted to the mark with deionized water. A Merck mixed metal standard (M6) was used as a certified reference to ensure the accuracy of analyses.

Samples were analysed for As and Cd using an Agilent 7500ce (Agilent Technologies, CA, USA) inductively coupled plasma-mass spectrometry (ICP-MS) (with octopole reaction system), employing an RF forward power of 1540 W and reflected power of 1 W, with argon gas flows of 0.81 L min^−1^ and 0.21 L min^−1^ for carrier and makeup flows, respectively. The instrument was operated in spectrum multi-tune acquisition mode, and three replicate runs per sample were employed. Each mass was analysed in fully quant mode (three points per unit mass).

The following isotopes were monitored: ^75^As, ^89^Y and ^111^Cd. ^103^Rh was added as internal standard at a concentration of 20 μg kg^−1^. ^111^Cd was analysed in no-gas tune, ^75^As was analysed using helium tuning to remove any polyatomic interferences, while the internal standards ^103^Rh and ^89^Y were analysed in both modes.

A series of standards were prepared by serial dilution of a 1000 mg L^−1^ stock solution with HNO_3_ 2% (v/v) (Merck KGaA, Darmstadt, Germany). The calibration curve fit (at least five standard concentrations) was of R^2^=0.999 in all cases. The mean concentration in blank digests was 0.07 μg L^−1^ for As and 0.05 μg L^−1^ for Cd. The detection limit was 0.01 μg L^−1^ for both metals. All analyses were performed in triplicate for each pot and are reported on a dry weight (DW) basis.

### X-ray absorption spectroscopy

In order to determine the speciation in which the metals exist in plant biomass as a basis for understanding uptake and detoxification mechanisms (Adediran et al. 2015; Adele et al. 2018), we analysed roots and leaves using X-ray absorption spectroscopy on beamline B18 at Diamond Light Source. Due to limited beamtime, we chose to compare biomass from the domestic genotype 1 and wild genotype 3 only. Plant material was dried in an oven at 70 °C for 72 h and finely ground for speciation analysis. This effectively open-air drying is likely to have affected the oxidation state of As in plant tissues from those in fresh biomass, rendering speciation outcomes unreliable. Thus, although we have As data (which in fact shows both trivalent and pentavalent As), the remainder of this contribution will focus on Cd only. Spectra were collected using QEXAFS in a liquid nitrogen cryostat to reduce sample damage, at the Cd K-edge, using a Si 311 monochromator. Spectra were acquired in fluorescence mode by means of a 9-element solid-state Ge detector. The beamline energy was calibrated using a Cd foil (26711 eV), and data were collected up to 13 A^−1^ with 0.5 eV resolution. Consecutive spectra from the same point were examined for possible beam damage, and damage was minimal.

Spectra were analysed using the Demeter suite of programmes (Ravel and Newville 2005). XANES spectra were compared to freshly prepared Cd standard solutions (nitrate, phytate, cysteine, citrate, malate, and histidine), all prepared at 4 mM (pH 5 for Cd phytate and Cd Cysteine, pH 7 for the other standards) and held in polythene tubes. We then performed EXAFS analysis to assess the coordination environment of Cd in leaves and roots. Coordination numbers were changed manually and are estimated to be ±1 given the small useful data range. The goodness of the fit was estimated by calculating the residual *R* factor; *R* = Σ_i_ (experimental-fit)^2^/Σ_i_ (experimental)^2^. A lower *R* factor represents a better match between the fitted standard spectra and the sample spectrum (Terzano et al. 2008).

### Data analysis

The phytoextraction ability of cardoon plant was evaluated by calculating the metals yield (mg) in shoot dry biomass and the translocation factor (TF) of As and Cd as below:

metals yield (mg) in shoot dry biomass = the heavy metal concentration in the shoots multiplied by the total biomass weight at the end of experiment (Zhao et al. 2003; Adediran et al. 2015);

TF= the heavy metal concentration in shoot (mg kg^−1^)/the heavy metal concentration in roots (mg kg^−1^) at the end of experiment (Zhao et al. 2003; Adediran et al. 2015).

Differences in the growth of the plants (biomass and root length) and in element accumulation in different plant organs, genotypes and treatments were subjected to the Bartlett’s test for homogeneity of variance and then analysed using factorial analysis of variance (ANOVA), using CoStat software (CoHort software, Montenery, CA, USA). The means were statistically separated on the basis of Student–Newmann–Keuls test when the ‘F’ test of ANOVA for treatment was significant at least at 0.05 probability level. Significance was accepted at p≤0.05 level (Snedecor and Cochran 1989).

## Results and discussion

### Plant growth parameters

To assess the effects of Cd and As on plant growth, the total plant biomass and the roots and shoots biomass were measured after harvest. The variation of the biomass allocation among organs is considered a useful parameter to the selection of plants to be used in phytoremediation applications (Iori et al. 2017). The statistical analysis showed that sampling time did not influence the plant growth parameters studied; therefore, we compared the data at the end of the experiment (45 days) only.

During the growth cycle, no visible toxicity symptoms (death or defoliation) were observed, and all the leaves were similar to the control. Despite the presence of metals, the treated plants continued to grow and survived until the end of the trial, but the biomass production was different, depending on genotype and metals type and amount added to the soil. Moreover, the results showed that growth might be stimulated, when the heavy metal dose is low; this finding is in line with the results reported by Feng et al. (2018) on Cd and Cu tolerance and bioaccumulation in *Sesuvium portulacastrum*.

**Fig. 1:**
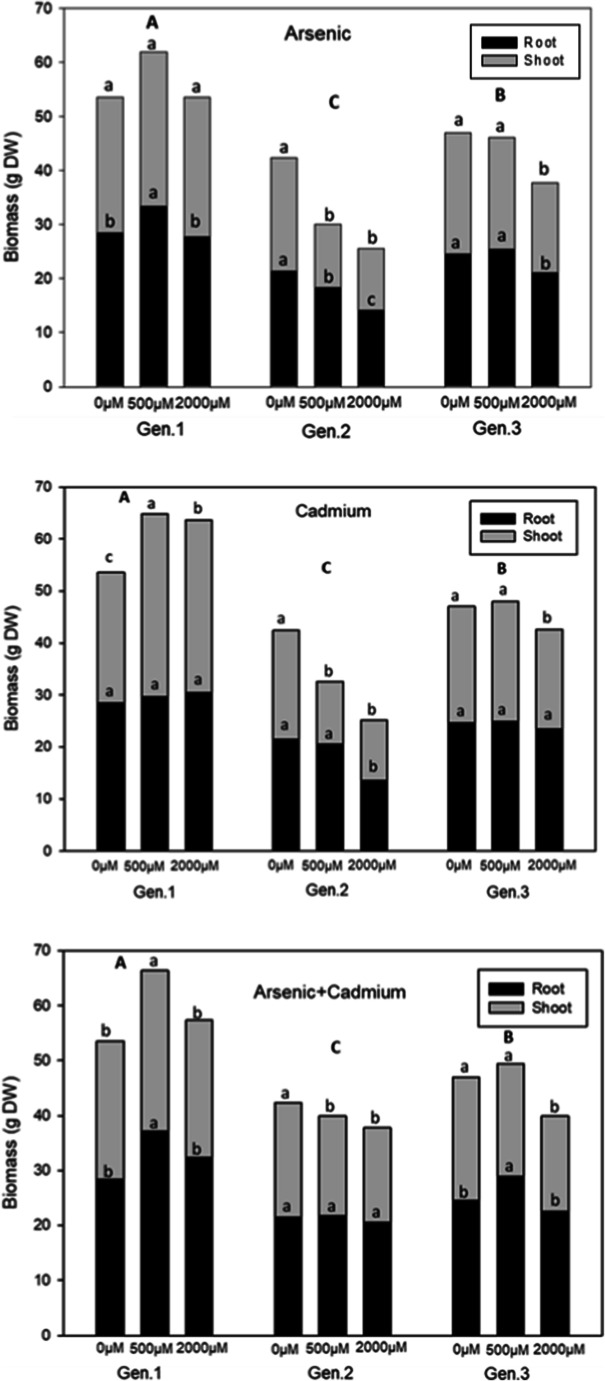
Root and shoot biomass of cardoon genotypes in response to As, Cd and As+Cd treatments at 45 days after contamination. Values are expressed as mean of biological replicates (n = 3). Different uppercase letters indicate statistically significant differences among the biomass on different levels contaminations among the genotypes. Different lowercase letters indicate statistically significant differences among the biomass on different levels contaminations among the same genotype (p≤0.05)

Figure 1 reports the dry biomass of different parts of plants; control plants showed a similar trend in biomass partitioning, allocating most of the dry biomass in the roots with a mean value of 24.81 g DW. The highest biomass was recorded for Gen.1, and the treated plants showed mostly a higher total biomass than control plants. In particular, the plants treated with As showed a behaviour significantly different from controls, but only at low As concentration: the growth of plants was stimulated with an increase in the total biomass mostly allocated in the roots with values of 33.45 g DW for As500 and 28.44 g DW for control. The same trend was observed in As+Cd treatments at low concentration with an increment of the total plant biomass mostly allocated in the roots, indicating a strong Cd and As tolerance when the two contaminants were together. In particular for As500+Cd500, the value of the roots biomass was 37.10 g DW, statistically different from the control (28.44 g DW). This phenomenon was considered one of the many interesting paradoxes related to As toxicity (Woolson et al. 1971; Carbonell-Barrachina et al. 1997, 1998; Miteva 2002; Garg and Singla 2011; Finnegan and Chen 2012). This stimulating effect at low As concentrations was from a direct interaction of As with plant metabolism or with soil plant nutrients (Finnegan and Chen 2012; Guarino et al. 2018). Most plants considered tolerant possess mechanisms to retain much of their As burden in the root (Finnegan and Chen 2012), which allows them to avoid As toxicity, with growth benefit deriving from As stimulation of inorganic phosphate (Pi) uptake (Tu and Ma 2003; Finnegan and Chen 2012). In fact, arsenate is taken up through the transport system of Pi transporter (PHT) proteins (Ullrich-Eberius et al. 1989; Meharg and Macnair 1990, 1991, 1992; Wu et al. 2011) in As hyperaccumulators (Wang et al. 2002; Tu and Ma 2003), As-tolerant non-hyperaccumulators (Meharg and Macnair 1992; Bleeker et al. 2003) and As-sensitive non-accumulators (Abedin et al. 2002; Esteban et al. 2003). From this interaction, as reported in a study on *Pteris vittata* L., phosphate likely substantially increased plant biomass and arsenate accumulation by alleviating arsenate phytotoxicity (Tu and Ma 2003).

By contrast, with Cd treatments, there was an increase of the plant biomass, mostly of the shoots, in both concentrations with values of 35.24 g DW for Cd500, 33.25 g DW for Cd2000 and 28.44 g DW for control. The same trend is seen when cadmium was in combination with arsenic but only for As500+Cd500 with a value of 29.26 DW.

Raccuia and Melilli (2007) have reported significant differences among wild and cultivated cardoon genotypes for aboveground biomass yield; in this work, we wanted to study the differences of biomass production of different genotypes even in the presence of heavy metal(loid)s. Results showed that wild cardoon had a biomass production lower than domestic one and Gen.3 showed a higher biomass production than Gen.2. It is possible that Gen.3 had developed adaption strategies to defend itself against environmental stresses due its provenance from highly polluted area. In particular, under As500+Cd500 treatment, the results of roots biomass production showed a value of 28.92 g DW that was statistically different from the control (24.57 g DW). One mechanism that plants use for As detoxification is the reduction of arsenate, As(V), to arsenite, As(III); the complexation of As(III) with phytochelatins (PCs), produced from the plants; and the sequestration to vacuoles (Li et al. 2015).

Regarding shoot biomass, Gen.3 showed a behaviour similar to the control at low concentrations and for all treatments studied. In fact, the low concentrations were favourable for plant growth, while high concentrations caused inhibition effect. However, the growth of Gen.2 was inhibited, showing a decrease of biomass, at both concentrations and for all treatments studied. In particular the lowest value of 11.56 g DW was under As2000 that was statistically different from the control plants (20.97 g DW).

**Fig. 2:**
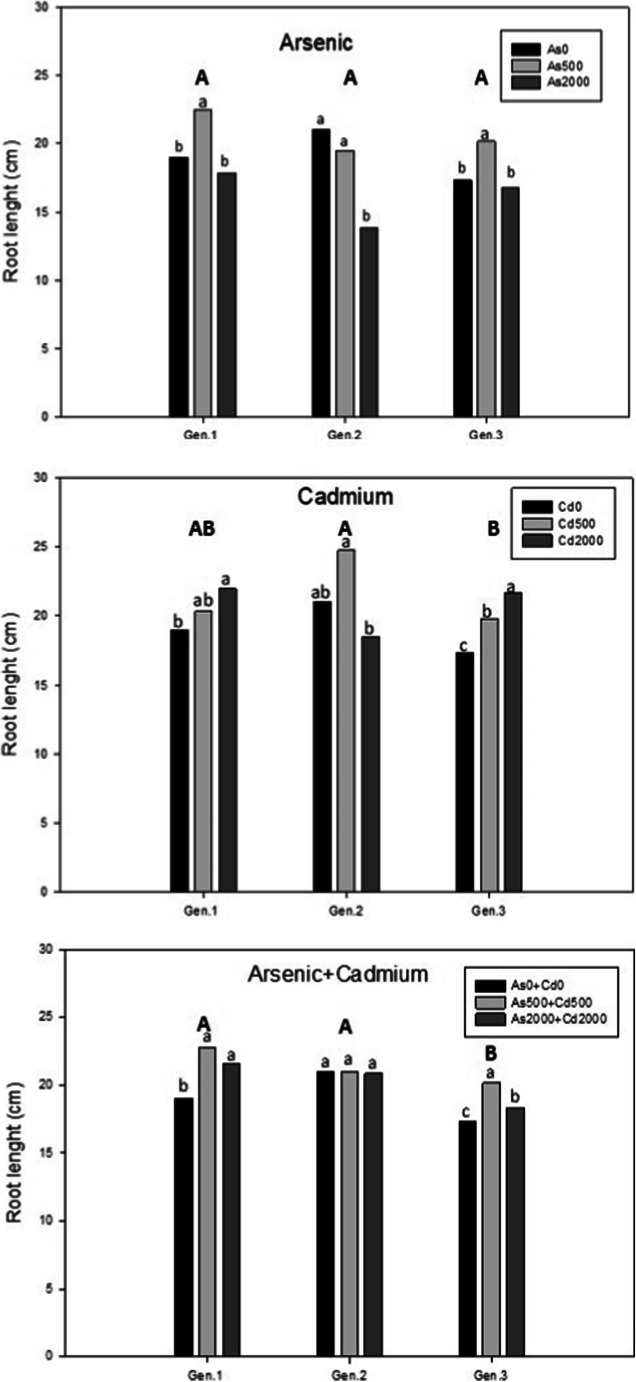
Root length of cardoon genotypes in response to different As, Cd and As+Cd treatments at 45 days after contamination. Values are expressed as mean of biological replicates (n = 3). Different uppercase letters indicate statistically significant differences among the root length on different levels contaminations among the genotypes. Different lowercase letters indicate statistically significant differences among the root length on different levels contaminations among the same genotype (p≤0.05)

Root elongation was influenced by metal concentrations (Fig. 2). In particular, regarding As treatment, there were no statistically significant differences among genotypes, but the toxic metal stress stimulated the root length, at low concentration only, for Gen.1 and Gen.3 with values of 22.50 cm and 20.17 cm, respectively, statistically different from the control plants (19.00 cm and 17.33 cm, respectively). The root length of Gen.2 decreased with increasing metal concentration, especially at As2000 with a value of 13.83 cm compared to the control (21.00 cm). Using Cd treatment, Gen.3 was significantly different from the other genotypes in showing stimulation of root length for both concentrations, with values of 19.83 cm for Cd500 and 21.67 for Cd2000.

In the presence of both metals, the results showed a significant stimulation of root length at both concentrations for Gen.1 and Gen.3, but the highest value (22.75 cm) was only at As500+Cd500 for Gen.1. Indeed, statistical analysis showed there was no stimulation in Gen.2.

### Heavy metals accumulations

**Fig. 3:**
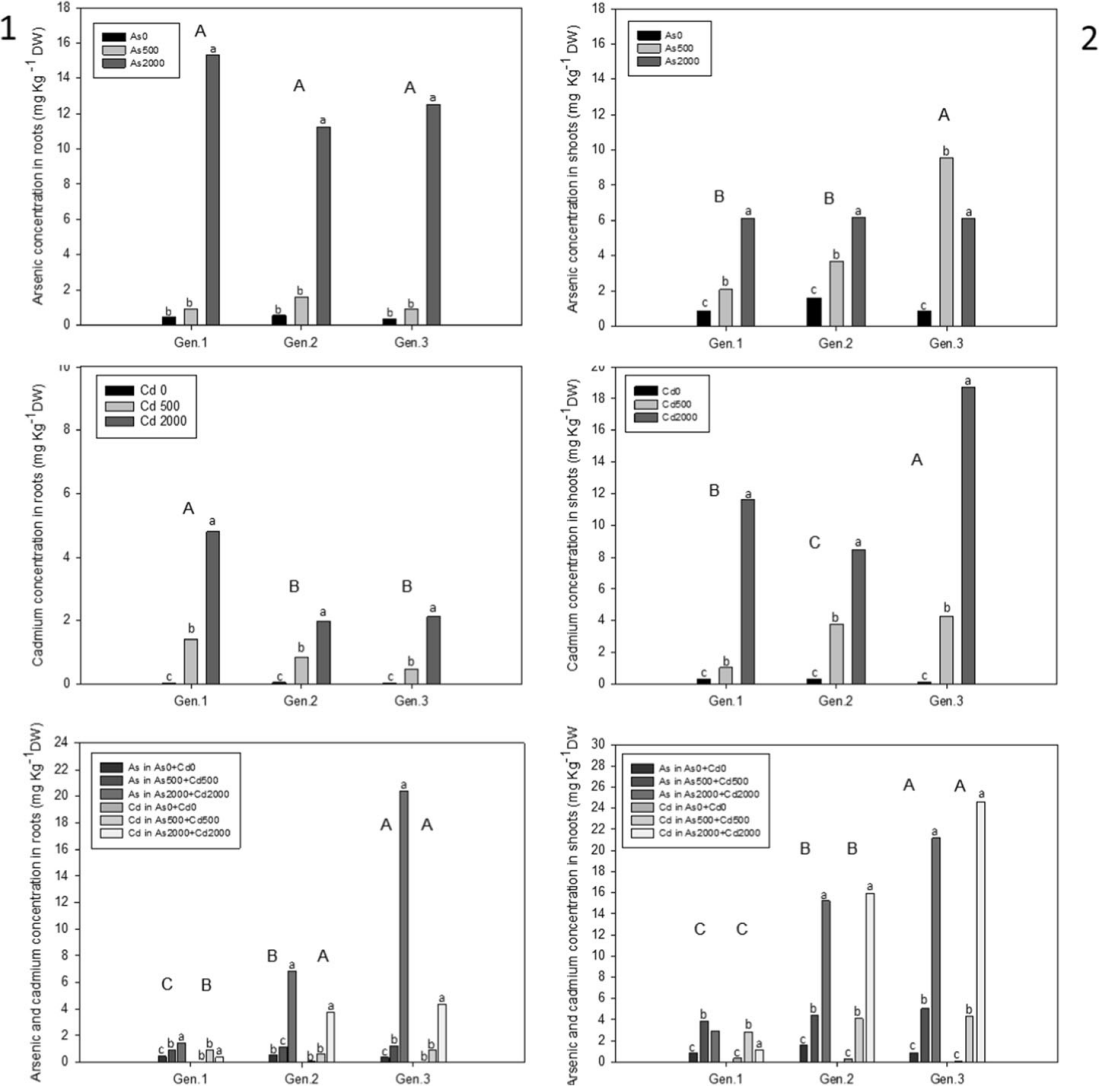
Concentration of As, Cd and As+Cd in roots (1) and shoots (2) of different cardoon genotypes, spiked with As and Cd, alone or in combination, at the end of experiment. Values are expressed as mean of biological replicates (n = 3). Different uppercase letters indicate statistically significant differences among the roots and shoots concentration on different levels contaminations among the genotypes. Different lowercase letters indicate statistically significant differences among the roots and shoots concentration on different levels contaminations among the same genotype (p≤0.05)

Arsenic and cadmium accumulations in the plants were analysed at different concentrations and at different times of exposure. The statistical analysis showed that the times of exposure did not influence the parameters studied; for this reason, only the accumulations of As and Cd at the end of the experiment were considered. The results showed that for all genotypes, As accumulated mainly in the roots (Fig. 3-1). Moreover, the arsenic root concentrations increased significantly with increasing As contamination in the soil. In particular, under As2000 μM, the As concentrations in roots were 15.32 mg kg^−1^ in Gen.1, 11.22 mg kg^−1^ in Gen.2 and 12.50 mg kg^1^ in Gen.3 (Fig. 3-1), whereas under As500 μM, the corresponding values were 0.91mg kg^−1^, 1.58 mg kg^−1^ and 0.90 mg kg^−1^. Our work supports the observation of Llugany et al. (2012) that showed that, regardless of the form of supplied As, cardoon plants accumulated As mainly in the roots, consistent with immobilization of the As in root cells. Our results are also consistent with Gupta et al. (2008) who found that As was preferentially concentrated in roots relative to shoots in chickpeas (*Cicer arietinum L*.), interpreted to be due to enhanced production of thiols in roots. Thus, although most studies show that As is translocated to shoots, other studies have shown that the actual distribution can depend on a variety of factors, including plant species, pH, redox state of the soil and microbial activity (Abbas et al. 2018).

Regarding shoots accumulation, as shown in Fig. 3-2, Gen.3 showed a different behaviour, compared to the other genotypes, with a higher capacity of accumulation of arsenic in leaves at As500.

Cadmium accumulation in cardoon roots showed the same behaviour as arsenic accumulation and increased significantly with the increase of the Cd concentration in the soil. The highest Cd concentration in roots was 4.79 mg kg^−1^ in Gen.1 under Cd2000 μM (Fig. 3-1). However, Cd accumulation was lower than that of arsenic in roots for all genotypes. In contrast with As, Cd concentrations in shoots were higher than those in roots, and the plants accumulated higher levels of Cd under the highest concentration of metal in the soil. The highest value of 18.72 mg kg^−1^ DW was found under Cd2000 μM in Gen.3 (Fig. 3-2). Although other studies generally report higher concentrations of Cd in roots relative to shoots, our observations are consistent with studies of Capozzi et al. (2020) and Arena et al. (2017) that showed that Cd on cardoon plants exhibited the highest values of the translocation factor (TF) indicating higher concentration in shoots than in roots. According to Chaney and Giordano (2015) and Alloway (1995), Cardoon’s efficient translocation for Cd occurs via transporters of Ca^2+^, Fe^2+^, Mg^2+^, Cu^2+^ and Zn^2+^ ions, into aerial parts of plants through this interaction with the available nutrient elements (Nazar et al. 2012; Arena et al. 2017). Also, cauliflower and sunflower planted in moderately Cd-contaminated soil showed enhanced Cd uptake in shoots and low accumulation in roots (Ma et al. 2021; Zehra et al. 2020a). It has been suggested that in shoots, the detoxification of Cd occurs through the synthesis of sulphur-rich organic acids, such as glutathione and phytochelatins, which sequestered Cd into vacuoles (De la Rosa et al. 2004; Huguet et al. 2012).

In their study, Llugany et al. (2012) showed that As accumulation was higher in plants grown in the presence of Cd than in those exposed to As alone. This means that the presence of Cd increased the ability of the plants to absorb As and translocate it to shoots, suggesting the potential ability of cardoon plants for synergic phytoextraction of Cd with other metals. Our results are consistent with earlier study of Sahito et al. (2021) that evaluated the arsenic accumulation in sunflower accessions in the presence of mercury, and they found the highest concentration of As in the above-ground parts of plants. In fact, in our work, the concentrations of both metals were always greater than those in treatments of As and Cd alone. Furthermore, we showed that for both metals, there were significant differences between genotypes studied, with the highest accumulation of metals in Gen.3 (Fig. 3, 1–2).

Moreover, our results are in accordance with the study of Pappalardo et al. (2020) that showed that *sylvestris* activated genes associated with transport of contaminant and which are involved in the synthesis of strong chelators that bind the metals in a non-toxic form. In particular, a*ltilis* and *sylvestris* plants treated with Cd and As expressed genes for phytochelatin synthase (PS), natural resistance of macrophage (NRAMP3), heavy metal ATPase (HMA), inorganic phosphate transport (PHT), ABCC transporter and zinc and iron protein (ZIP) that are involved in abiotic stress response in model plants. The same authors also showed that NRAMP3, ZIP11, ABCC and PHT genes, that usually are activated in accumulator model plants, under Cd or As stress were activated also in wild cardoon, but not in the domestic one.

### Cadmium speciation and coordination in biomass

**Fig. 4:**
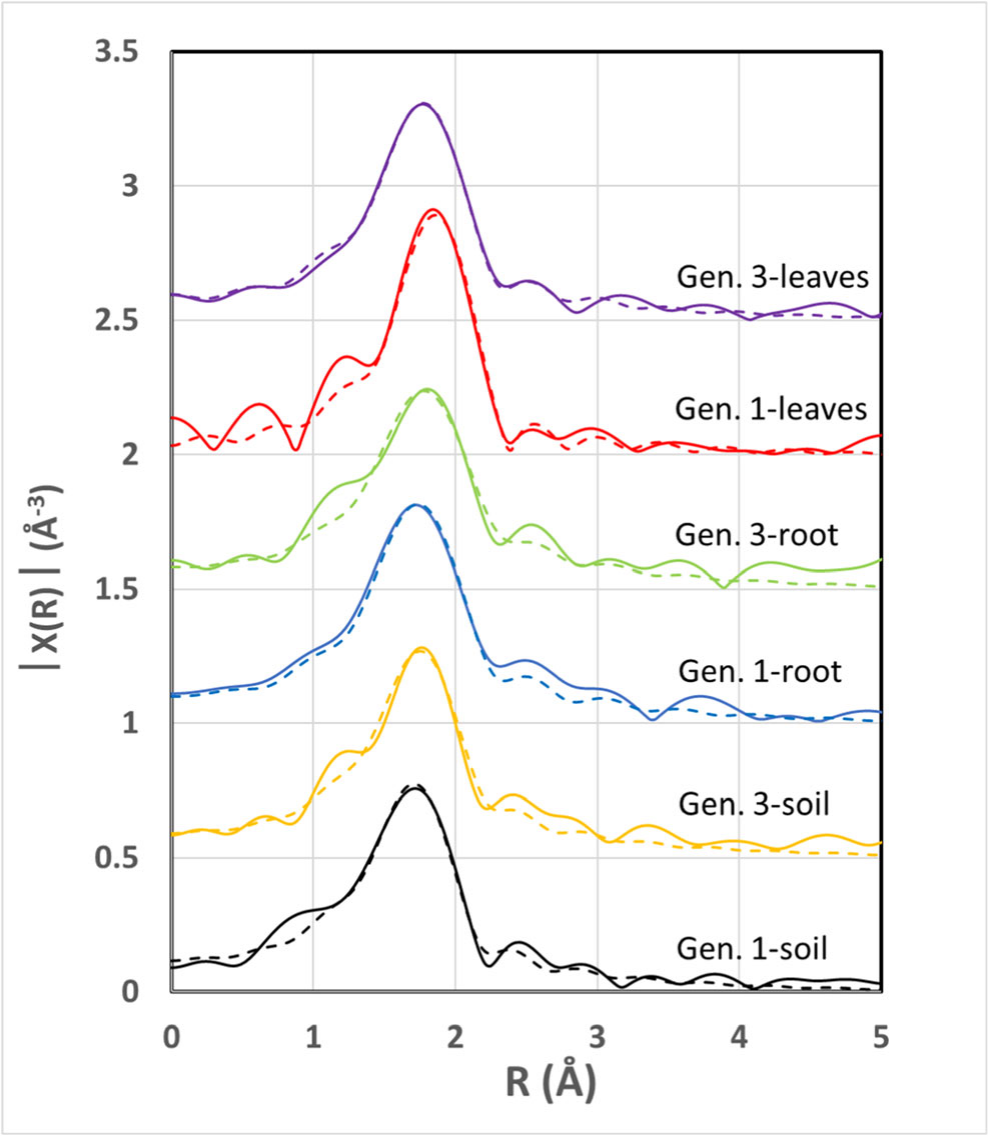
Fourier transforms of the k^2^-weighted EXAFS data for Cd K-edge data of soil, root and leave spectra comparing the two genotypes analysed for this study (Experimental data solid line, fit data dotted line. The y-axis has been manually offset by 0.5 for each sample for clarity.)

Comparison of standards and samples showed no variation in the XANES (probably due to the large core hole lifetime of 7.3 eV at the Cd K-edge), precluding reasonable assessment of Cd speciation distribution among plant tissues. Nevertheless, Fourier transformed data showed a shift in the main feature around 1.7 Å between roots and leaves (Fig. 4). Hence, we performed EXAFS fitting of the soil, root and leaf data in order to investigate possible changes in coordinating atoms around Cd. Due to the limited range of the EXAFS data (k ≤10 Å ^-1^), only first shell coordination was possible.

In soil samples, Cd was always coordinated to 6 oxygen atoms at a distance of 2.28±0.01 Å (Table 1), consistent with the Cd-O distance in CdCO_3_ (Boyanov et al. 2003). Cd was also coordinated to 6 oxygen atoms in roots of both genotypes, with the same bond distance. Differences between genotypes emerged in the coordination of Cd in leaves. In the domesticated genotype 1, coordination was dominated by Cd-S, with 4 sulphur atoms around each Cd atom at a distance of 2.45±0.01 Å. Addition of oxygen atoms did not improve fit to the data. By contract, Cd-O bonding dominated Cd coordination in leaves of the wild genotype 3, with 5 oxygen atoms and one sulphur atom around each Cd. There were no apparent differences between samples treated with Cd only and those treated with a mixture of Cd and As.

**Table 1:** Cd co-ordination in soil, root and leaf samples of Gen.1 and Gen.3 as determined by EXAFS modelling of Synchrotron X-ray Spectroscopic data

Genotype	Sample	Atom	N° of atoms	Interatomic distance/Å	Debye Waller Factor/Å^2^	k fit range (Å^-1^)	R value
Gen.1	Soil 6_30	O	6	2.27 (0.01)	0.008 (0.001)	3–10	0.007
	Root 86	O	6	2.28 (0.02)	0.009 (0.001)	3–9	0.011
	Leaves 86	S	4	2.45 (0.01)	0.007 (0.001)	3–9	0.018
Gen.3	Soil 24	O	6	2.28 (0.02)	0.009 (0.003)	3–10	0.018
	Root 24	O	6	2.30 (0.02)	0.010 (0.003)	3–9	0.018
	Leaves 24	O	5	2.29 (0.01)	0.009 (0.002)	3–9.5	0.003
		S	1	2.50 (0.03)	0.012 (0.007)		

We noted above that the wild Gen.3, sourced from an industrial area (assumed to be contaminated), accumulated more of each metal (Cd and As) than either the wild Gen.2, sourced from a clean area, or the domesticated Gen.1. Furthermore, Gen.3 grew better than Gen.2, although it produced lower biomass than Gen.1. We attributed this behaviour of Gen.3 to possible development of adaptive mechanisms that enable it to tolerate metal toxicity during its growth on contaminated soil. Using similar techniques (EXAFS), Isaure et al. (2015) showed that Cd in non-metal resistant species of *Arabidopsis halleri* was predominantly coordinated to sulphur atoms whereas in metal-resistant species, Cd was coordinated to sulphur and oxygen atoms. They postulated that coordination to oxygen-containing ligands (possibly organic acids) was responsible for metal tolerance in these phenotypes. Our results are consistent with this interpretation, if we consider Gen.1 to be non-tolerant to Cd, and that it responds to Cd uptake by producing sulphur-containing ligands to complex and translocate Cd into shoots. Such a mechanism is consistent with our previous studies on Zn toxicity and uptake in *Brassica juncea* when inoculated with bacteria (Adediran et al. 2015; Adele et al. 2018).

### Phytoextraction efficiency and translocation factor of arsenic and cadmium

**Fig. 5:**
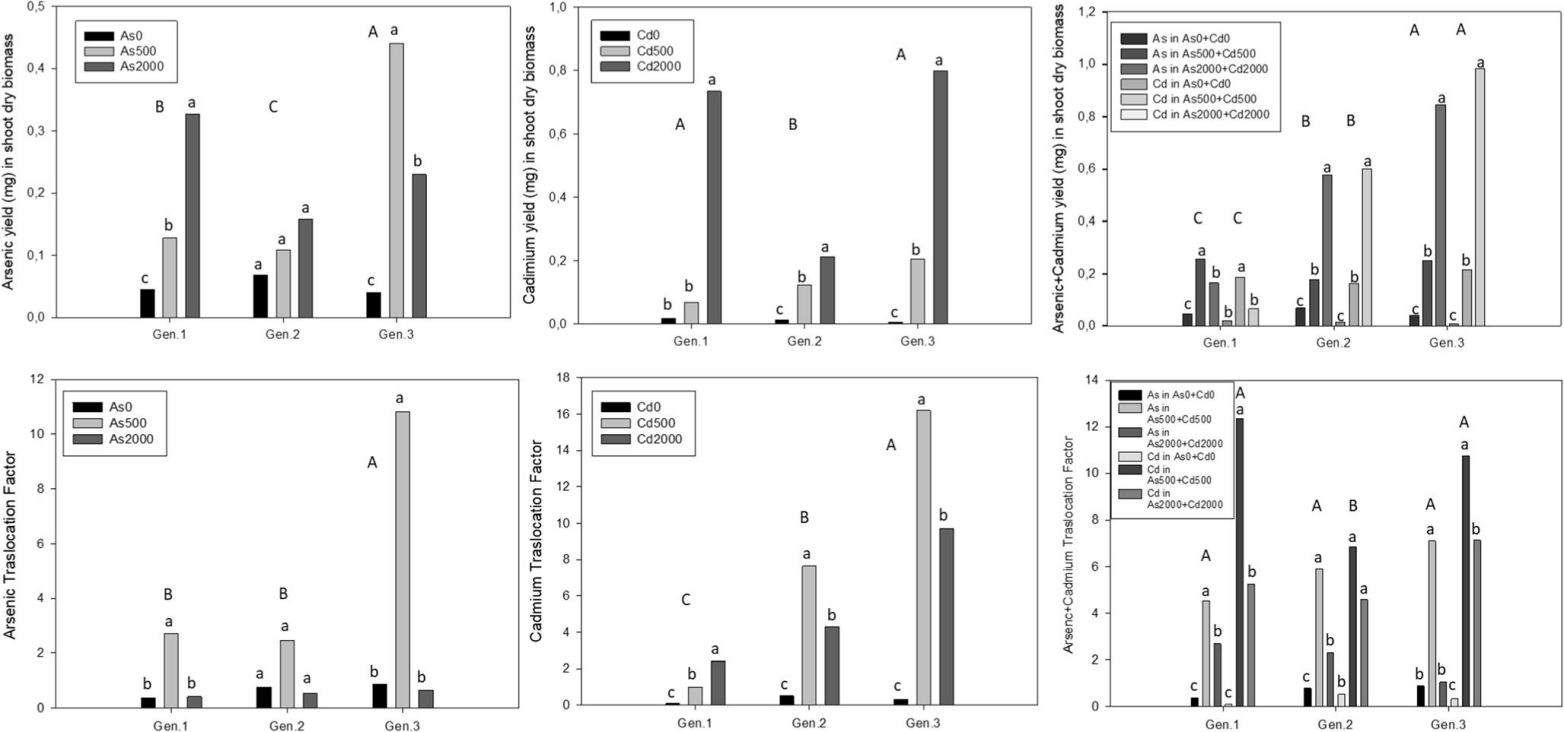
Arsenic, cadmium and arsenic+cadmium yield in shoot dry biomass and translocation factor under different concentrations in cardoon, at the end of experiment. Values are expressed as mean of biological replicates (n = 3). Different uppercase letters indicate statistically significant differences among the metals concentration on different levels contaminations among the genotypes. Different lowercase letters indicate statistically significant differences among the metals concentration on different levels contaminations among the same genotype (p≤0.05)

As and Cd yield in shoot dry biomass and translocation factor (TF) were measured (Fig. 5). To understand the phytoextraction ability of cardoon plants, the association of high biomass production and the ability to accumulate contaminants in its tissues were assessed by calculating the metal yield in shoot dry biomass. The best phytoextraction was achieved in cadmium treatment, where the yield of metal accumulation in shoot increased significantly with the increase of the Cd concentration in the soil. The highest values of 0.735 mg and 0.798 mg were found under Cd 2000 μM in Gen.1 and Gen.3, respectively. When the metals were in association, the highest values of 0.983 mg in dry shoot was found for Gen.3 in Cd2000 treatment that was statistically different from the control (0.007 mg).

Moreover, for effective toxic metal phytoextraction, TF is an important parameter for assessing the ability of a plant to translocate the absorbed metal from the root to harvestable aerial biomass, and it should be greater than 1.0 (Wei and Chen 2006; Adediran et al. 2015). In this work, the best results were mostly achieved in the low concentrations of all treatments, but the highest TF (16.19) was in plants of Gen.3 in Cd500 treatment. The results confirmed that Cd showed more phytoextraction (higher in shoots than roots) across treatments and genotypes, suggesting the possibility to use cardoon plants to remediate Cd-contaminated soil through phytoextraction techniques.

## Conclusion

All cardoon genotypes accumulated As mainly in the roots, indicating the immobilization of this metal in root cells. By contrast, cadmium was accumulated especially in leaves; that means that cardoon plants had a good translocation ability to transfer Cd from roots to shoots, with translocation apparently effected by sulphur-rich ligands, possibly cysteine, glutathione or phytochelatins, based on EXAFS analysis. The interaction effect of As+Cd has increased the resistance of plants to these metals, allowing the plants to survive, even in presence of high concentrations of both metals. Furthermore, the accumulation of metals was higher in plants exposed to co-contamination of As and Cd than that of plants under As or Cd alone. Also, cardoon, under As+Cd contamination, translocated more As from root to shoots/leaves. Lastly, comparing the cardoon genotypes studied, the results demonstrated that *C*. *cardunculus* L. var. *sylvestris*, A14CT (Gen.3), collected from polluted soil, was the one that accumulated high levels of both contaminants, adapting mechanisms that enable it to tolerate metal toxicity during its growth on contaminated soil. It suggests its use in future works to remediate soils from these toxic elements as with both ability of phytoextraction and phytostabilization, thanks to its good tolerance of both heavy metal(loid)s.

For this reason, it would be useful to continue the trials with the selected genotype 3, with the aim to test for more years its remediation efficiency in polluted soils, taking advantage, at the same time, the use of these marginal lands for the biomass production for sustainable bioenergy purposes.

## Data Availability

All data generated or analysed during this study are available from the corresponding author on reasonable request.
